# Pathways of influence on health-promoting lifestyles in older adults with coronary heart disease: a cross-sectional study

**DOI:** 10.3389/fcvm.2025.1652844

**Published:** 2025-08-29

**Authors:** Zhaoxia Tian, Yue Zhang, Hongmei Li, Xiaochun Lv, Quanyi Wang, Weiwei Tian

**Affiliations:** ^1^ Department of Nursing, Fenyang College of Shanxi Medical University, Fenyang, Shanxi, China; ^2^ Department of Cardiovascular Medicine, Fenyang Hospital of Shanxi Province, Fenyang, Shanxi, China

**Keywords:** coronary heart disease, health literacy, social support, self-efficacy, health-promoting lifestyle

## Abstract

**Background:**

The global demographic shift toward aging populations has precipitated a significant rise in coronary heart disease (CHD) prevalence among older adults, positioning health promoting lifestyles as a critical component of healthy aging initiatives. Despite increasing recognition of psychosocial factors in chronic disease management, the complex pathways influencing health promoting lifestyles adoption in elderly CHD patients remain insufficiently understood. Current research lacks theoretically grounded investigations examining how cognitive and social determinants interact to shape health behaviors in this vulnerable population.

**Objective:**

Based on social cognitive theory, this study aims to explore the influencing factors of health-promoting lifestyles in elderly patients with CHD and the path relationships among them.

**Methods:**

A cross-sectional study was conducted from a tertiary care hospital. Based on social cognitive theory, the Health Promoting Lifestyle Scale and its related research tools were adopted. Structural equation modeling (SEM) elucidated direct and indirect relationships between variables, supported by correlation analyses and model fit statistics.

**Results:**

236 eligible elderly patients with CHD participated in this study. Analysis of the results showed that health literacy, perceived social support, and general self-efficacy had a direct positive effect on health-promoting lifestyles, health anxiety has a direct negative effect on health-promoting lifestyles, and perceived social support, self-efficacy, and health anxiety had significant indirect effects on health-promoting lifestyles.

**Conclusion:**

This study establishes an social cognitive theory-informed framework where psychosocial resources and cognitive appraisals synergistically influence health behaviors in older adults. The centrality of self-efficacy as a mediator highlights its role in translating environmental supports into sustainable lifestyle changes. Based on the pathway relationships among various factors identified in the study, clinical nursing can construct a comprehensive nursing model. During the nursing process, a comprehensive assessment of the patients’ various indicators should be conducted, and personalized nursing plans should be formulated according to the assessment results. It is necessary to organically integrate aspects such as improving health literacy, strengthening social support, enhancing self-efficacy, and alleviating health anxiety to provide patients with all-round nursing services.

## Introduction

Coronary Atherosclerotic Heart Disease (CHD), commonly referred to as coronary heart disease, is a leading non-communicable disease impacting global health and a significant public health concern worldwide (1). According to the Summary of the China Cardiovascular Health and Disease Report 2024, statistical analysis of data from the “China Cardiovascular and Cerebrovascular Events Surveillance” program in 2023 revealed that the crude incidence rate of acute myocardial infarction (AMI) among residents aged ≥18 years was 87.6 per 100,000. The incidence of AMI showed a rapid upward trend with increasing age (2). Cardiovascular diseases are the primary cause of mortality. The main cause of death in patients is associated with cardiovascular diseases (3). Elderly patients often present with comorbidities such as hypertension, diabetes, and hyperlipidemia, making the elderly a high-risk population for CHD. This poses a severe threat to their health. The incidence of CHD increases annually, particularly in individuals aged 60 and older, with a prevalence rate of 27.8%. The age group most affected is between 65 and 84 years (4). The high prevalence of CHD in the elderly exacerbates the burden on families, patients, and society, creating challenges for the Healthy China initiative. Therefore, the health of elderly CHD patients is a critical public health issue that demands significant societal attention (4).

Health-promoting lifestyles represent self-managed health behaviors individuals adopt to achieve holistic well-being, playing a crucial role in optimizing health and preventing disease (5). High adherence to health-promoting lifestyles can reduce disease incidence, alleviate symptoms, promote physical and mental health, and enhance quality of life and life satisfaction (6). For elderly patients with chronic diseases, health-promoting lifestyles are essential for maintaining function and improving quality of life (7). In terms of functional maintenance, resistance exercise combined with high-quality protein diets can delay sarcopenia (8), while activities like Tai Chi can improve balance and joint flexibility, reducing the risk of falls (9). Regarding quality of life, regular exercise and mindfulness meditation can alleviate pain symptoms and improve sleep quality (10). Furthermore, health-promoting lifestyles offer significant socioeconomic benefits, reducing hospitalization rates and delaying disability onset in elderly patients, thereby alleviating the long-term care burden on families and society. Overall, health-promoting lifestyles shift from the traditional disease-centered medical model to a life-centered approach, utilizing multi-dimensional interventions to help elderly patients with chronic diseases achieve both independent living and dignity, representing a core strategy for secondary prevention and elderly health management (11).

Elderly patients with coronary heart disease face multi-dimensional challenges in changing health behaviors, including physiological decline, social role transitions, and psychological adaptation, with these factors interacting to form complex mechanisms (12). Physiologically, the significant decline in activity endurance and weakened balance and coordination in the elderly are associated with issues such as articular cartilage wear and bone loss, which not only limit daily activities and exercise but also increase the risk of falls (13). Social role transitions also pose challenges for elderly coronary heart disease patients. The loss of social status and social networks may be associated with retirement, and a shift in family roles from economic provider to care recipient, potentially causing feelings of loss, uselessness, and guilt, which can negatively impact health behavior motivation, even the avoidance of treatment or refusal to change lifestyles by people to reduce family burdens is also associated with this. These challenges collectively hinder health behavior changes.

**Figure 1:**
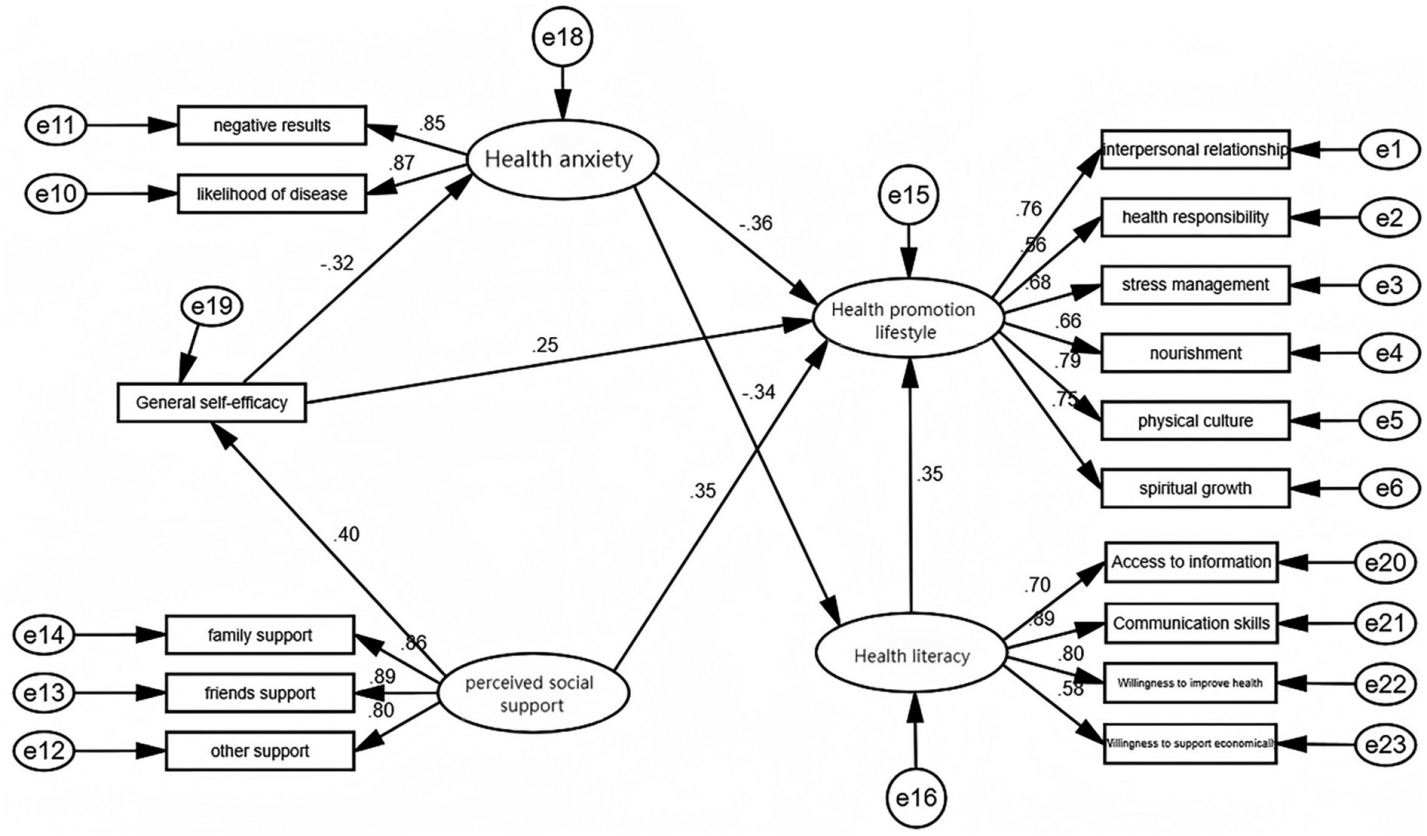
Path mechanism diagram of the impact of perceived social support on health – promoting lifestyles.

Social Cognitive Theory (SCT) posits that patients’ cognition, behavior, and environmental factors interact and influence each other, with their synergy promoting autonomy in daily life (14), helping patients understand how to promote positive behavior changes through role models, self-regulation, and social support (15). Existing research often focuses on observable variables that individuals cannot or find difficult to change, such as age, gender, and marital status. However, other unobservable variables, such as beliefs, abilities, and attitudes, also influence health behavior changes in chronic disease patients. Currently, few studies combine Social Cognitive Theory to comprehensively consider variables like health literacy, self-efficacy, and socio-demographic and disease-related factors to explore the impact of health-promoting lifestyles. This study hypothesizes that perceived social support can directly improve the level of health-promoting lifestyles in elderly patients with coronary heart disease, or achieve this effect indirectly by enhancing self-efficacy (Figure 1).

This study employed path analysis to construct a cognitive social theory model, aiming to investigate the relationships among health literacy, health anxiety, social support, self-efficacy, and healthy lifestyles in elderly patients with coronary heart disease (CHD). By analyzing these path relationships, we sought to comprehensively understand the health status of elderly CHD patients, providing a theoretical basis and practical guidance for improving their health outcomes. The findings of this research offer scientific evidence for developing effective interventions, with practical implications for enhancing health management and promoting healthy aging in this population.

## Methods

### Study design

This study employed a cross-sectional design. From August 2024 to March 2025, elderly coronary heart disease patients were recruited as research subjects through convenience sampling in the Department of Cardiology of a Grade III, Class A hospital in Shanxi Province. After obtaining informed consent from the patients, this study was conducted in the form of a questionnaire survey.

### Participants

Inclusion criteria: (1) meeting the diagnostic criteria for coronary heart disease (16); (2) age ≥60 years; (3) clear consciousness and good communication skills; (4) voluntary participation.

Exclusion criteria: (1) combined with severe complications such as liver and kidney; (2) those with severe mental or psychological illnesses; (3) those who refuse to cooperate with the questionnaire survey. (4) patients with a history of percutaneous coronary intervention (PCI), coronary artery bypass grafting (CABG), or heart failure.

According to the sample size estimation method (17), 5–10 times the number of variables were included. This study included 7 variables of general information, 6 dimensions of health-promoting lifestyle, 2 dimensions of health anxiety, 1 dimension of general self-efficacy, and 4 dimensions of health literacy for patients with chronic diseases, totaling 23 variables. Considering a 20% sample loss, the sample size should be 138–276 cases. This study ultimately surveyed 236 patients.

### Data collection

The core variables of this study are perceived social support, self-efficacy, health literacy, and health-promoting lifestyle.

### General information questionnaire

Designed by the researchers, including age, gender, education level, marital status, place of residence, family history, and monthly income.

### Health-Promoting Lifestyle Profile (HPLP)

The Health-Promoting Lifestyle Profile was developed by Cao Wenjun et al. (18) in 2016 to measure the level of health-promoting lifestyle of the participants. The questionnaire includes 6 dimensions and 40 items, covering health responsibility, physical activity, interpersonal relationships, spiritual growth, stress management, and nutrition. A 4-point Likert scale was used to collect participant responses. Scores ranged from 1 point to 4 points, from “never” to “always” with a total score ranging from 40 points to 160 points. The higher the score, the higher the level of health-promoting lifestyle of the participants. In this study, the Cronbach's alpha coefficient of the questionnaire was 0.928.

### Perceived Social Support Scale (PSSS)

The Perceived Social Support Scale was developed by Huang Li et al. (19) in 1996 to measure the degree of perceived social support of the participants. The questionnaire includes 3 dimensions and 12 items, covering family support, friend support, and other support. A 7-point Likert scale was used to collect participant responses. Scores ranged from 1 point to 7 points, from “strongly disagree” to “strongly agree” with a total score ranging from 12 points to 84 points. The higher the score, the higher the degree of perceived social support of the participants. In this study, the Cronbach's alpha coefficient of the questionnaire was 0.948.

### General Self-Efficacy Scale (GSES)

The General Self-Efficacy Scale was developed by Wang Caikang et al. (20) in 2001 to measure the self-efficacy of the participants. The questionnaire includes 10 items. A 4-point Likert scale was used to collect participant responses. Scores ranged from 1 point to 4 points, from “not at all true” to “exactly true” with a total score ranging from 10 points to 40 points. The higher the score, the better the general self-efficacy of the participants. In this study, the Cronbach's alpha coefficient of the questionnaire was 0.902.

### China Short Health Anxiety Inventory (CSHAI)

The China Short Health Anxiety Inventory was translated by Zhang Yuqun et al. (21) and developed by Salkovakis et al. (22) in 2002. This questionnaire comprises 2 dimensions and 18 items, assessing the fear of illness and the negative consequences of being ill. A 4-point Likert scale was employed to collect participant responses, ranging from “never” to “always”. Scores ranged from 0 point to 3 points. The total score ranges from 0 to 54, with higher scores indicating greater health anxiety. A score of 15 was used as the threshold for screening health anxiety. In this study, the Cronbach's alpha for this questionnaire was 0.962.

### Health Literacy Scale for Chronic Patients (HLSCP)

The Health Literacy Scale for Chronic Patients was developed by Sun Linhao (23) in 2012. This questionnaire includes 4 dimensions and 24 items, covering communication and interaction skills, willingness for economic support, information acquisition ability, and the willingness to improve health. A 5-point Likert scale was used to collect participant responses, ranging from “very difficult” to “not difficult”. Scores ranged from 1 point to 5 points. The total score ranges from 24 to 120, with higher scores indicating higher health literacy levels. In this study, the Cronbach's alpha for this questionnaire was 0.964.

### Data collection

Data for this study were collected through the Jinshuju platform, a reliable data collection platform in China, where researchers distributed online questionnaires. Prior to the survey, two investigators received professional and standardized training, ensuring they understood the item meanings, scoring methods, and precautions of the survey tools. Participants and their families were informed about the survey's purpose, significance, and questionnaire completion methods before the survey. They were provided with a QR code for the Jinshuju questionnaire, which they could complete independently. For those unable to complete the questionnaire themselves due to visual or educational limitations, the investigators read the questions and assisted with completion. The questionnaire took approximately 30 min to complete. The completeness of the questionnaires was checked on the spot to identify any omissions or non-compliant items, ensuring the validity and completeness of the questionnaires (24). A total of 260 questionnaires were distributed, with 236 valid questionnaires returned, resulting in an effective recovery rate of 90.7%.

### Statistical analysis

Data were exported from the Jinshuju platform to EXCEL for preliminary processing and then imported into SPSS 26.0 for descriptive statistics and statistical tests. Categorical data were described using frequencies and percentages, while numerical variables that met the normal distribution were described using means and standard deviations. The estimation method is the maximum likelihood method. Harman's single-factor test was used to examine the common method bias of the questionnaire. Pearson correlation analysis was used to explore the correlations between variables. The SEM model was constructed using Amos 24.0 to examine the path relationships between variables. The mediating effect was tested using the Bootstrap method (with 5,000 repeated samplings), and the 95% confidence interval (95% CI) was adopted as the criterion for significance. When the 95% confidence interval does not contain 0, it indicates that the mediating effect is statistically significant (*P* < 0.05).

## Results

### General sociodemographic characteristics of the participants

A total of 236 participants were included, with 128 males and 108 females. The mean age of the participants was 70.92 ± 6.47 years. Approximately half of the participants had a high school education level (48.3%), and 89.4% were married. Regarding income, 64.8% of the participants had an income above 3,000 RMB. Most participants resided in rural areas (61.4%), and most had a family history of cardiovascular disease (58.1%). More specific details are presented in Table 1.

**Table 1:** Univariate analysis of sociodemographic data on health-promoting lifestyles.

Variable		Frequency (%)	Mean (standard deviation)	*t*/*F* value	*P*-value	Effect size
Age	60–69	96 (40.7)	114.00 (19.92)	1.976	0.141	0.017
	70–79	106 (44.9)	108.37 (20.99)			
	>80	34 (14.4)	112.06 (19.09)			
Gender	Male	128 (54.2)	110.90 (20.95)	3.263	0.072	0.031
	Female	108 (45.8)	111.54 (19.78)			
Education	Primary education	59 (25.0)	102.56 (20.00)	25.94	*P* < 0.001	0.251
	high school education	114 (48.3)	106.46 (20.28)			
	bachelor's degree	54 (22.9)	127.33 (7.33)			
	Master's degree	9 (3.8)	130.89 (8.39)			
Marital status	Married	211 (89.4)	111.5 (20.43)	0.472	0.493	0.147
	Unmarried/divorced/widowed	25 (10.6)	108.52 (20.16)			
Place of residence	Urban	91 (38.6)	111.52 (20.39)	0.002	0.964	0.026
	Rural	145 (61.4)	110.99 (20.44)			
Family history of cardiovascular disease	Yes	137 (58.1)	112.04 (20.29)	0.077	0.781	0.1
	No	99 (41.9)	110.01 (20.54)			
Monthly income	≤3,000	83 (35.2)	101.98 (20.12)	29.33	*P* < 0.001	0.738
	>3,000	153 (64.8)	116.19 (18.77)			

### Univariate analysis of sociodemographic characteristics on health-promoting lifestyles

Univariate analysis was employed to assess the impact of sociodemographic characteristics on health-promoting lifestyles. The total score for health-promoting lifestyles in elderly patients with coronary heart disease was 111.19 ± 20.38. Univariate analysis revealed significant correlations between health-promoting lifestyles and educational level (*F* = 25.94, *P* < 0.001) and monthly income (*F* = 29.33, *P* < 0.001) in elderly patients with coronary heart disease (Table 1).

### Correlation analysis among variables

The analysis indicated positive correlations between health-promoting lifestyles and health literacy, self-efficacy, and perceived social support, while a negative correlation was observed with health anxiety. Furthermore, self-efficacy was positively correlated with perceived social support and negatively correlated with health anxiety. Health literacy showed positive correlations with perceived social support and self-efficacy, and a negative correlation with health anxiety. See Table 2 for details.

**Table 2:** The results of Pearson correlation analysis among variables.

Variable	Health promotion lifestyle	General self-efficacy	Health literacy	Health anxiety	Perceived social support
Health Promotion Lifestyle	1				
General self-efficacy	.518**	1			
Health literacy	.557**	.260**	1		
Health anxiety	−.549**	−.277**	−.335**	1	
perceived social support	.571**	.376**	.289**	−.405**	1

**P < 0.01.

### Pathway analysis of mediating effects

As indicated in Table 3, the regression analysis revealed that general self-efficacy exerted a direct positive predictive effect on perceived social support (*β* = 6.252, *P* < 0.001) and health-promoting lifestyle (*β* = 7.708, *P* < 0.001). Perceived social support directly and positively predicted health-promoting lifestyle (*β* = 0.38, *P* < 0.001), while it indirectly and negatively predicted health anxiety (*β* = −0.278, *P* < 0.001). Furthermore, health anxiety directly and negatively predicted health-promoting lifestyle (*β* = −0.363, *P* < 0.001).

**Table 3:** Regression analysis of chain mediation effect.

Outcome variable	Predictor variable	Significance of regression coefficient				Overall fit index				*F*
		*β* (standardized)	(95% CI)		*t*	*P*	*R*	*R* ^2^	Adj.*R*^2^	
Perceived social support	General self-efficacy	6.252	3.963	8.542	5.381	*P* < 0.001	0.443	0.197	0.186	18.934**
	Education	3.669	1.565	5.773	3.435	0.001				
	Monthly income	3.487	−0.043	7.017	1.946	0.053				
Health anxiety	perceived social support	−0.278	−0.402	−0.154	−4.434	*P* < 0.001	0.487	0.237	0.224	17.918**
	General self-efficacy	−2.205	−4.524	0.114	−1.874	0.062				
	Education	−3.656	−5.717	−1.596	−3.497	0.001				
	Monthly income	−3.608	−7.007	−0.21	−2.092	0.038				
Health Promotion Lifestyle	Health anxiety	−0.363	−0.499	−0.227	−5.275	*P* < 0.001	0.781	0.61	0.602	71.949**
	Perceived social support	0.38	0.245	0.515	5.563	*P* < 0.001				
	General self-efficacy	7.708	5.264	10.151	6.215	*P* < 0.001				
	Education	6.851	4.64	9.062	6.106	*P* < 0.001				
	Monthly income	6.864	3.276	10.452	3.77	*P* < 0.001				
Health literacy	Health Promotion Lifestyle	0.522	0.39	0.655	7.755	*P* < 0.001	0.562	0.316	0.307	35.694**
	Education	1.856	−1.357	5.069	1.138	0.256				
	Monthly income	2.576	−2.459	7.612	1.008	0.314				
Perceived social support	Health Promotion Lifestyle	0.403	0.314	0.492	8.904	*P* < 0.001	0.571	0.327	0.318	37.505**
	Education	−0.15	−2.31	2.01	−0.137	0.891				
	Monthly income	−0.008	−3.393	3.377	−0.005	0.996				

**P < 0.001.

### Construct a structural equation modeling (SEM)

Harman's single-factor test was used to examine common method bias. The test results showed that the variance contribution rate of the first principal component was 25.97%, which is lower than the 40% threshold. This indicates that the overall common method bias of the questionnaire is not serious and falls within an acceptable range.

A structural equation modeling was constructed using Amos 24 to test the path relationships. The goodness-of-fit test results of the constructed SEM model are as follows: Root Mean Square Error of Approximation (RMSEA) = 0.069 < 0.08, and Goodness of Fit Index (GFI) = 0.908 > 0.9; among the incremental fit indices, Comparative Fit Index (CFI) = 0.944 > 0.9, Tucker–Lewis Index (TLI) = 0.931 > 0.9, and Incremental Fit Index (IFI) = 0.944 > 0.9. All the measured fit indices meet the standard criteria, indicating that the constructed SEM model has a good fit and is relatively reliable.

As indicated in Table 4, this study employed a bootstrapping method with 5,000 iterations to compute the 95% confidence intervals, thereby further validating the mediation effects. The results demonstrate that the impact of perceived social support on health-promoting lifestyles is mediated by general self-efficacy. Furthermore, a chain mediation effect was observed, where general self-efficacy and health anxiety sequentially influenced health-promoting lifestyles. Additionally, health anxiety directly affected health-promoting lifestyles and indirectly influenced them through health literacy.

**Table 4:** Bootstrap (5,000 iterations) mediation effect test.

Pathway relationship	Dependency	Efficiency value	95% CI		*P*	Effect proportion
			LLCI	ULCI		
A→B→C	Indirect effect	0.046	0.025	0.075	<0.001	22.4%
	Direct effect	0.159	0.090	0.243	<0.001	77.6%
	Total effect	0.205	0.136	0.293	<0.001	100.0%
D→E→C	Indirect effect	−0.025	−0.043	−0.013	<0.001	25.3%
	Direct effect	−0.074	−0.109	−0.046	<0.001	74.7%
	Total effect	−0.099	−0.135	−0.069	<0.001	100.0%
A→B→D→C	Indirect effect	0.021	0.009	0.040	<0.001	11.7%
	Direct effect	0.159	0.090	0.243	<0.001	88.3%
	Total effect	0.180	0.112	0.264	<0.001	100.0%

A, perceived social support; B, general self-efficacy; C, Health Promotion Lifestyle; D, health anxiety, E, health literacy.

## Discussion

This study investigates the influencing factors of health-promoting lifestyles in elderly coronary heart disease (CHD) patients from the perspective of social cognitive theory, exploring the pathways of each influencing factor. The research yielded three key findings. Firstly, perceived social support demonstrated both direct and indirect positive effects on health-promoting lifestyles, while self-efficacy also showed direct and indirect positive effects. Conversely, health anxiety exhibited direct and indirect negative effects on health-promoting lifestyles. Health literacy was found to have a direct positive effect on health-promoting lifestyles. Secondly, the impact of perceived social support on health-promoting lifestyles can be mediated through general self-efficacy, or indirectly through a chain relationship involving general self-efficacy and health anxiety. Thirdly, health anxiety can also indirectly influence health-promoting lifestyles through health literacy.

Perceived social support has a direct or indirect positive effect on health-promoting lifestyles. As a chronic disease affecting the cardiovascular system, CHD often necessitates lifelong medication, causing significant physical suffering and impacting the normal lives of patients and their families, severe psychological trauma is also associated with this (25). High levels of perceived social support can buffer and protect patients when facing the health challenges and life threats of CHD, encouraging them to actively seek health-promoting lifestyles, thereby alleviating their fear of the disease. This study found that self-efficacy is the most significant factor in indirect effects, the effect size is 0.046, which is lower than that in Reyes et al.'s study (26). This may be related to the fact that all participants in this study are elderly, whose lifestyles are less malleable. Harvey et al. (27) showed that extensive social support from family and friends can help patients suppress the onset of adverse symptoms, prompting them to actively construct health management behavior patterns, thereby enhancing self-efficacy and gradually alleviating anxiety about the disease. Wang Fen et al. (28) demonstrated that the more social support resources patients receive after PCI, the more they can overcome inertia in exercise and promote the establishment of post-operative health-promoting behaviors. Li Weijuan et al. (29) showed that the stronger the self-efficacy of nurses in health behaviors, the higher the level of health-promoting lifestyles. Conversely, when nurses lack the motivation to engage in health behaviors, it is difficult for them to practice a healthy lifestyle. Du et al. (30) found that elderly patients with strong self-efficacy are more inclined to participate in health behaviors based on their health knowledge when managing daily activities. It is evident that social support has a positive effect on health-promoting lifestyles. If patients receive less perceived social support and do not receive effective interventions, it will affect their ability to establish regular healthy habits. Therefore, medical staff should help CHD patients obtain more social support pathways, actively improve patients’ levels of perceived social support, and proactively care for patients. This can be achieved through sharing patient cases, promoting popular science articles, and watching popular science videos to improve patients’ self-efficacy (31), thereby helping patients face the disease with a positive lifestyle.

Self-efficacy demonstrates both direct and indirect positive effects on health-promoting lifestyles. This study reveals that self-efficacy exerts a positive and significant influence on health-promoting lifestyles, and it can also indirectly affect these lifestyles through health anxiety. High levels of self-efficacy can improve patients’ physical and mental health, treatment adherence, and disease coping abilities (32), thereby enhancing their self-management capabilities and encouraging them to implement sustainable health behavior interventions. Numerous studies have confirmed (30, 33, 34) a positive correlation between self-efficacy and health-promoting lifestyles, elderly individuals with high self-efficacy are more confident in maintaining their health and can better adhere to health behaviors. Coronary heart disease (CHD) patients may experience reduced self-efficacy due to the long duration of the disease, heavy economic burden, or insufficient social support, thereby affecting their health-promoting lifestyles. Therefore, it is recommended that relevant departments, hospitals, and communities focus on CHD patients, providing them with convenient follow-up appointments, medication discounts, and assisting them in proactively adopting strategies related to health behaviors.

Health anxiety exhibits both direct and indirect negative effects on health-promoting lifestyles. Path analysis results indicate that health anxiety directly and negatively predicts health-promoting lifestyles and can also indirectly affect these lifestyles by influencing health literacy. The direct effect is more significant than the indirect effect, indicating that when CHD patients worry about the threat of the disease and fear its adverse consequences, the negative emotions of patients towards their own condition are related to this kind of anxiety, thereby negatively affecting their health-promoting lifestyles. This suggests that healthcare professionals should pay attention to patients’ health anxiety. They can guide patients to alleviate anxiety and enhance their confidence in overcoming the disease through health knowledge dissemination (35) digital health interventions (36), and other methods, thereby improving their health-promoting lifestyle capabilities.

Health literacy demonstrates a direct positive effect on health-promoting lifestyles. The results of this study indicate that health literacy directly and positively predicts health-promoting lifestyles, consistent with other research (37). Individuals with high health literacy (38) possess strong beliefs and sufficient abilities to acquire and utilize information; conversely, those with low health literacy may experience an increased risk of mortality. Therefore, healthcare professionals should emphasize the dissemination of health information. They can strengthen multi-department collaboration, mobilize patient participation, develop personalized educational measures based on individual patient circumstances, enhance patients’ health literacy, and promote the translation of knowledge into behavior.

Health literacy did not play a mediating role in the “social support→lifestyle” pathway, which differs from the findings of Shih et al. (39). This may be because the impact of social support on lifestyle can bypass health literacy and exert a direct effect. Specifically, the influence of social support on lifestyle may rely more on direct behavioral drive or emotional motivation rather than the improvement of health literacy at the cognitive level. Meanwhile, the role of health literacy itself is limited by behavioral execution conditions and information relevance, and may not have been fully captured due to limitations in the research design. In subsequent studies, more attention should be paid to types of support that directly empower behaviors, such as peer companionship and resource provision, rather than solely relying on the improvement of health literacy.

### Limitations

This study holds value in exploring the path analysis of factors influencing health-promoting lifestyles in elderly patients with coronary heart disease (CHD), yet it presents certain limitations. Initially, lifestyle data rely on participants’ subjective scale results, which inherently involve measurement bias. Due to social desirability effects and memory errors, the lifestyles reported by participants may differ from their actual behaviors. Although the study controlled for bias through methods such as training investigators and on-site verification of questionnaire completeness, subjective reporting remains difficult to avoid entirely. In the future, cross-validation can be conducted in combination with objective indicators (such as physiological test data and behavioral records in medical records) to reduce measurement bias. Secondly, the study employed convenience sampling, precluding the analysis of how health-promoting lifestyles are influenced by different types of CHD or co-existing underlying diseases. Future research should consider refining the classification of CHD patients to investigate these specific influences. In addition, this study was conducted in only one hospital, which has issues such as insufficient sample size and limited sample representativeness. Future multi-center, large-sample surveys are needed to further verify this conclusion. Thirdly, the study exclusively focused on elderly CHD patients, excluding middle-aged and young adults with CHD. Consequently, caution should be exercised when interpreting the study's findings, as they primarily reflect the circumstances of elderly CHD patients. Fourth, this study did not include clinical endpoint indicators, such as clear disease-related outcomes like readmission rates, myocardial infarction, and stroke, and only focused on intermediate variables or subjective indicators such as lifestyle and health literacy. Fifth, as this study adopted a cross-sectional design rather than a longitudinal design, it was unable to clarify the temporal sequence between variables, which may further affect the inference of causal relationships. Although this study included social demographic variables such as educational level and monthly income as control factors, there may be unincluded confounding factors (such as patients’ cultural customs, family care resources, details of past disease history, etc.). These factors may indirectly affect health-promoting lifestyles by influencing health literacy or social support, leading to residual confounding in the path analysis results. Future studies can further control confounding effects by expanding the scope of included variables or adopting methods such as propensity score matching.

## Conclusion

This research provides an in-depth exploration of the factors influencing health-promoting lifestyles in elderly CHD patients, elucidating the pathways of various influencing factors. Notably, the study identified the positive impacts of perceived social support, health literacy, and self-efficacy on health-promoting lifestyles. Therefore, clinicians should focus on patients’ levels of perceived social support, providing humanistic care to patients, promoting interactions between patients and their families, and enhancing patients’ self-efficacy to improve their health-promoting lifestyles. Understanding the complex pathways of factors influencing health-promoting lifestyles in elderly CHD patients will assist healthcare professionals in implementing targeted interventions in their practice, thereby improving the quality of care.

## Data Availability

The original contributions presented in the study are included in the article/Supplementary Material, further inquiries can be directed to the corresponding author.
